# Prostate Cancer with Intraluminal Inclusions: the Association of the Immunophenotype with Grade Score

**DOI:** 10.30699/ijp.2019.91400.1884

**Published:** 2019-08-01

**Authors:** Artem Pidddubnyi, Anatolii Romaniuk, Inna-Margaryta Radomychelski, Yuliia Moskalenko, Roman A. Moskalenko

**Affiliations:** 1 Department of Pathology, Medical Institute, Sumy State University, Sumy, Ukraine; 2 Department of Surgery and Oncology, Medical Institute, Sumy State University, Sumy, Ukraine

**Keywords:** Prostate cancer, Grade score, Immunohistochemistry, Prostatic calculi, Corpora amylacea

## Abstract

**Background & Objective:**

To study the immunophenotype of prostate cancer (PC) with the presence and absence of intraluminal inclusions (IIn), depending on the grade score.

**Methods:**

A total of 30 PC samples with IIn (group E) and 30 PC samples without them (group C) were studied. These groups were divided into 2 subgroups, depending on the grade of malignancy, which was determined according to the Gleason score as moderate and high-grade tumors. Macroscopic analysis, hematoxylin-eosin staining, immunohistochemistry (androgen receptors, p53 and Bax proteins, Hsp70 and Hsp90, CD68, VEGF, OSN, MMP-1) were used.

**Results:**

The expression level of VEGF was higher in the more differentiated tumors of the control group (*P*<0.01). Increased expression of prognostic-adverse markers p53 (in the presence of IIn, *P*<0.01) and MMP-1 (*P*<0.05) was observed. Also, a higher level of OSN expression was found in PC tissue with IIn (*P*<0.01) due to its participation in the processes of biomineralization. The expression level of CD68 and Bax protein was higher in the PC group with IIn (both *P*<0.01). Furthermore, Hsp90 had a significantly lower expression level in the PC of group E (*P*<0.05).

**Conclusion:**

the presence of IIn in the PC samples of group E promotes tissue remodeling with mechanical trauma, chronic inflammation, and fibrosis development. The presence of IIn in PC leads to the increase of OSN, CD68 and Bax expression and decrease of Hsp90 and VEGF expression. High expression of p53 and MMP-1 and low expression of OSN and VEGF was identified as a characteristic of high-grade tumors.

## Introduction

Prostate cancer (PC) is one of the most common causes of cancer-related deaths all over the world. According to the American Cancer Society, in 2010-2014 PC incidence and mortality rates were 118.2 and 19.5 cases per 100.000, respectively (1). The PC development and progression are closely related to the presence of chronic inflammation, which is associated with the presence of intraluminal inclusions (IIn) (prostatic calculi and amyloid corpuscles, or corpora amylacea) (2). The presence of IIn is also associated with an increased number of CD68 positive activated macrophages, the development of chronic pelvic pain syndrome and PC (3-4). Formation of IIn is a complex process, which involves the interaction of both tumor cells and tumor stroma. The remodeling of stromal components is manifested by the angiogenesis (the appearance of VEGF-positive cells) and the expression of stress tissue factors (Hsp70 and Hsp90), which have a tumorigenic effect and promote the development of bone metastases (5-6). Destruction of connective tissue (due to increased expression of MMP-1) causes migration and invasion of cancer cells, and this results in the development of the metastatic PC (7). Progression of PC is accompanied by increased levels of apoptosis (expression of the mutant p53 protein and Bax protein) and changes in sensitivity to androgens (decreased expression of receptors to androgens) (8,9). 

The course and prognosis of the malignant process is directly related to the grade score of the tumor tissue. The Gleason grading system is generally accepted to estimate the morphological status of PC (10). It has been shown that prognosis of the disease deteriorates and probability of metastasis (predominantly to the bones) increases significantly with increasing of PC grade score (11).

The aim of this study is to evaluate the immune-histochemical phenotype of prostate cancer with the presence and absence of intraluminal inclusions, depending on the grade score. 

## Materials and Methods

Samples of Prostate Cancer

The study was conducted on the biopsy material obtained during surgeries at the Sumy Regional Clinical Hospital and Sumy City Clinical Hospital 1. The selected PC samples were divided into two groups according to the presence/absence of IIn. In total, 30 PC samples with inclusions (experimental group, E) and 30 PC samples without inclusions (control group, C) were studied. The control and experimental groups were divided into 2 subgroups, depending on the grade score, which was determined according to the Gleason grading system: tumors with a moderate (C2 and E2) and high (C3 and E3) grade score. The groups C2 and E2 included tumors with 7-8 Gleason scores (class 2-4) and the groups C3 and E3 included tumors with 9-10 Gleason scores (class 5) (10).

The Ethics Commission

This study was approved by the ethics committee of the Medical Institute of Sumy State University (Proceedings 3/6; June 7, 2016).

Histology 

Biological material was fixed in 10% neutral buffer formalin solution for 24 hours. Subsequently, the material was dehydrated and paraffin embedded. Paraffin series were sliced at a thickness of 4 μm on a rotational microtome Shandon Finnesse 325 (Thermo Scientific, USA). Deparaffinized and rehydrated sections were stained with hematoxylin and eosin. 

Immunohistochemistry (IHC)

In summary, 4 µm-thick serial sections made from prepared paraffin blocks were applied to SuperFrost adhesive slides (Thermo Scientific, USA). The deparaffinized sections were subjected to demasking of the antigens by thermal treatment in citrate buffer (pH 6.0) at a temperature of 95-98°C. The UltraVision Quanto Detection System HRP Polymer (Thermo Scientific, USA) detection system was used for visualization of results. It includes reduction of the endogenous peroxidase activity with 3% hydrogen peroxide, blocking of non-specific background reaction with the "Ultra V Block", and enhancing with the "Primary Antibody Amplifier Quanto". Diaminobenzidine (DAB) was used as a chromogen. The following antibody panel was used (Thermo Scientific, USA): androgen receptors (AR), pro-apoptotic protein Bax and protein p53 (p53), matrix metalloproteinase 1 (MMP-1), vascular endothelial growth factor (VEGF), heat shock protein 86 kDa (Hsp90), heat shock protein of 70 kDa (Hsp70), CD68, and osteonectin (OSN) (Table 1).

**Table1:** Antibody panel for IHC

Antibody	Immunized Animal	Clone	Dilution	Expression pattern
AR	Rabbit	Polyclone	1:200	Nucleus
р53	Mouse	SP5	1:100	Nucleus
MMP-1	Rabbit	Polyclone	1:50	Cytoplasm
VEGF	Rabbit	Polyclone	1:200	Cytoplasm and membrane
Hsp90	Rabbit	Polyclone	1:100	Nucleus and cytoplasm
Hsp70	Rabbit	Polyclone	1:100	Nucleus and cytoplasm
Bax	Rabbit	Polyclone	1:100	Cytoplasm
CD68	Mouse	KP1	1:100	Cytoplasm
OSN	Rabbit	Polyclone	1:50	Cytoplasm

Morphometric study was conducted using the morphometric programs "SEO Scan ICH 285 AK-F IEE-1394" (Ukraine) and "Zen 2.0" (Carl Zeiss, Germany). The number of positive tumor cells was counted in fields with a diameter of 1000 μm. Photographing and storage of images were conducted using the digital imaging systems "SEO Scan ICH 285 AK-F IEE-1394" (Ukraine) and "ZEN" for Carl Zeiss microscopes (Germany). We used active (tissue with established positive and negative reactions) and passive control of results.

Statistical Analysis

The normality of all data sets was assessed by the Shapiro-Wilk test. In case of an abnormal distribution, a nonparametric method, the Mann-Whitney test, was used. In case of correct distribution, the data were compared using parametric Student’s *t*-test to determine the reliability of the difference. The results were considered statistically significant with a probability of more than 95% (*P*<0.05). The graphical representation of statistical analysis results was performed using the GraphPad Prism 7.04. 

## Results

Histological Structure of Tumor Tissue

**Figure 1:**
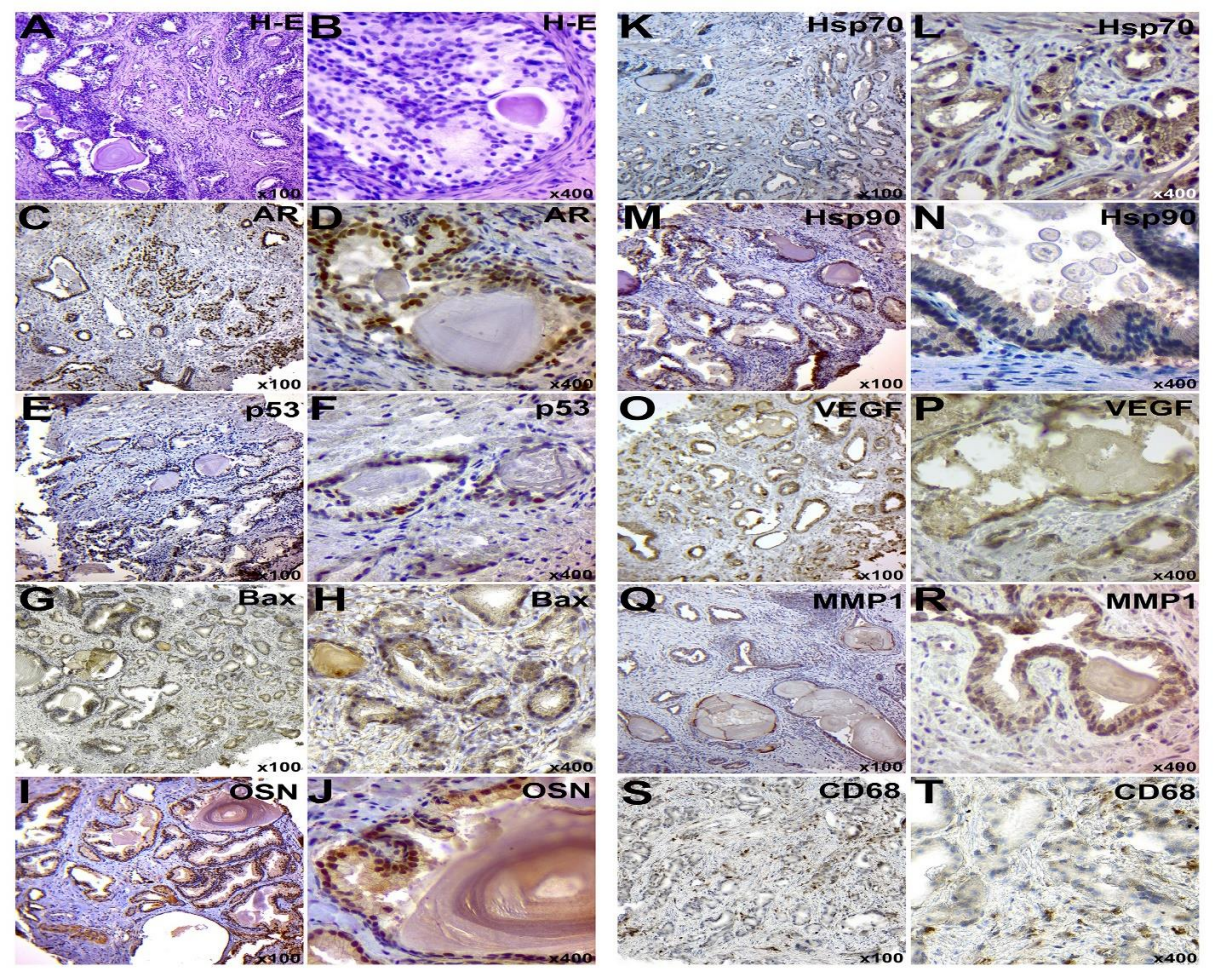
IHC examination of PC in subgroup E2: A-B – staining with hematoxylin and eosin; C-D – IHC detection of AR; E-F - IHC identification of p53 protein; G-H – IHC detection of p53; I-J – IHC of OSN expression; K-L – IHC detection of Hsp70; M-N – IHC detection of Hsp90; O-P – IHC detection of VEGF; Q-R – IHC detection of MMP-1; S-T – IHC detection of CD68+ positive cells. Chromogen – diaminobenzidine. Nuclei were counterstained with Mayer’s hematoxylin. The magnification is indicated in the lower right corner of each image

Histological analysis of the PC tissue of the experimental group with a moderate grade score (E2) showed the presence of glands, which were formed by atypical cells (Figs. 1a and 1b). These cells had hyperchromic nuclei, and the crybroid and pseudotrabecular structures were present. Well-developed stromal component was observed between tumor glands. Most glands had a lumen and were located close to each other.

**Figure 2:**
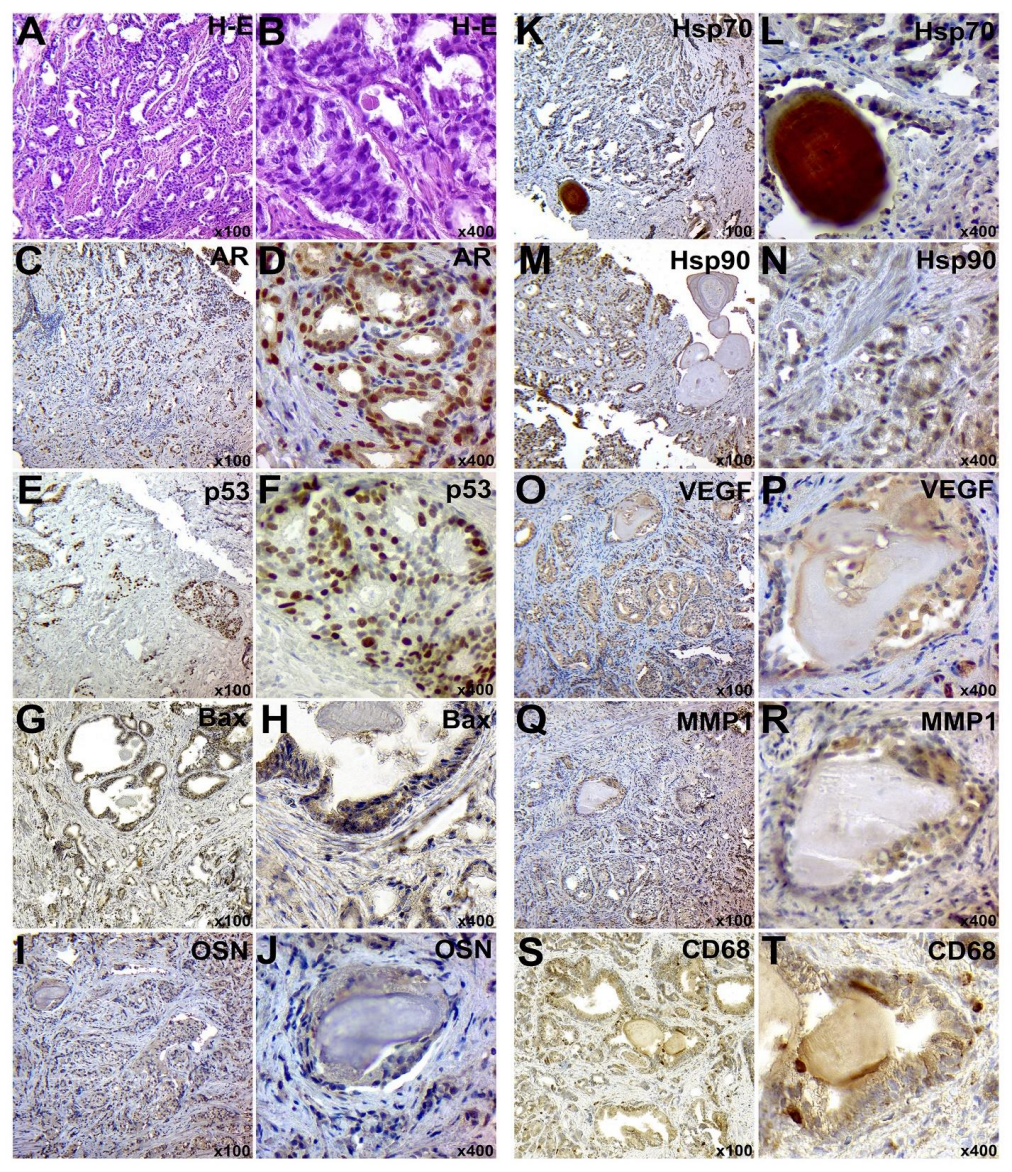
IHC examination of PC in subgroup E3: A-B – staining with hematoxylin and eosin; C-D – IHC detection of AR; E-F - IHC identification of p53 protein; G-H – IHC detection of p53; I-J – IHC of OSN expression; K-L – IHC detection of Hsp70; M-N – IHC detection of Hsp90; O-P – IHC detection of VEGF; Q-R – IHC detection of MMP-1; S-T – IHC detection of CD68+ positive cells. Chromogen – diaminobenzidine. Nuclei were counterstained with Mayer’s hematoxylin. The magnification is indicated in the lower right corner of each image

The PCs of group E3 were characterized by a significant violation of histological structure and simplification of tumor glands. These glands formed multiple chains and nests. Tissue of the tumors had a small amount of the stromal components (Figs. 2a and 2b). Most of the glands did not have a lumen. In addition, in majority of cases, the samples of this group were represented only by the tumor field.

Samples of the experimental group (E2 and E3) were characterized by the presence of IIn in the tumor glands. These inclusions were round-formed and repeated the shape of gland lumen. Prostatic calculi were characterized by dark brown color and a more homogeneous structure. Corpora amylacea had a layered structure and a dark pink color.

**Figure 3:**
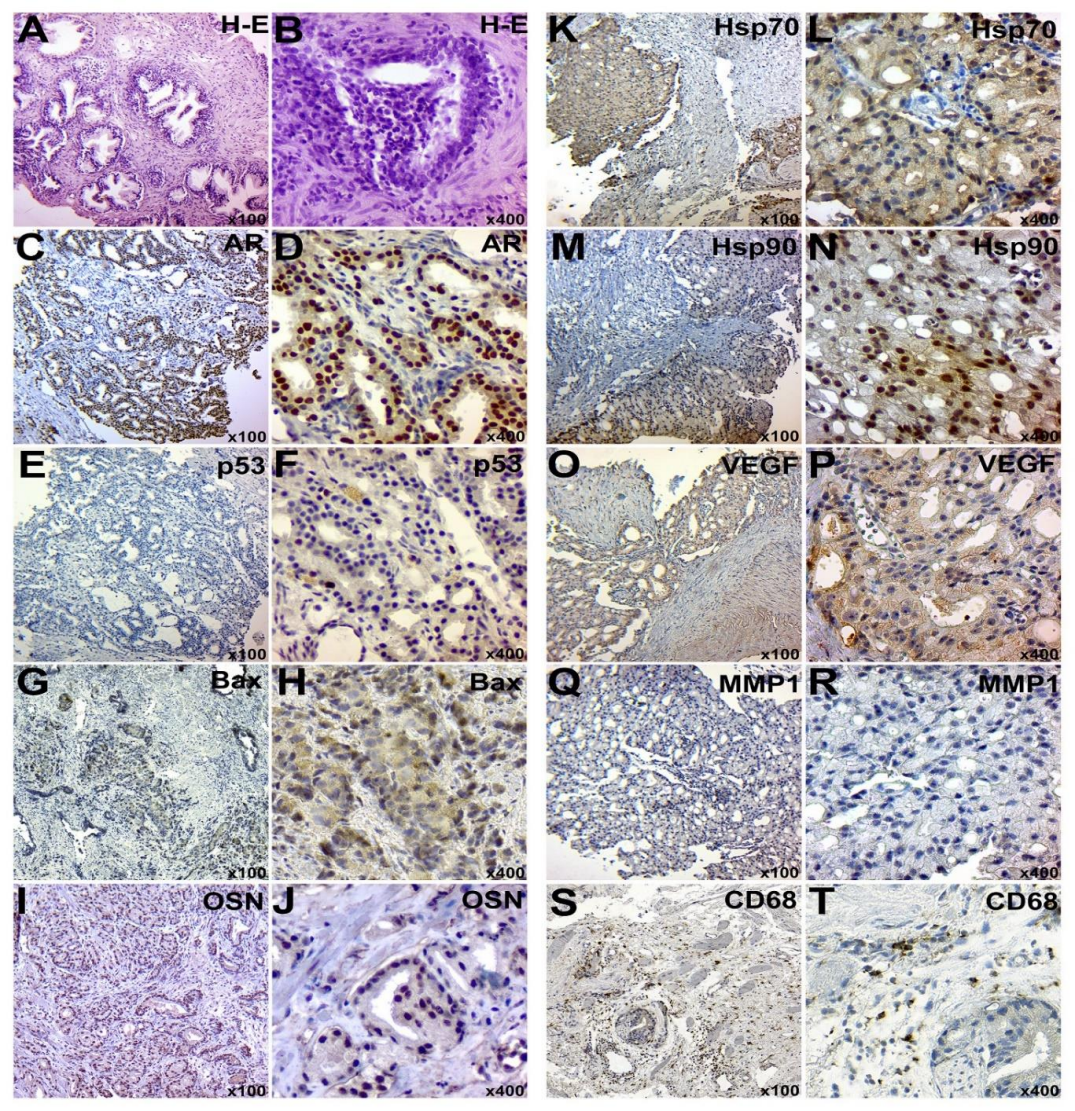
IHC examination of PC in subgroup C2: A-B – staining with hematoxylin and eosin; C-D – IHC detection of AR; E-F - IHC identification of p53 protein; G-H – IHC detection of p53; I-J – IHC of OSN expression; K-L – IHC detection of Hsp70; M-N – IHC detection of Hsp90; O-P – IHC detection of VEGF; Q-R – IHC detection of MMP-1; S-T – IHC detection of CD68+ positive cells. Chromogen – diaminobenzidine. Nuclei were counterstained with Mayer’s hematoxylin. The magnification is indicated in the lower right corner of each image

**Figure 4:**
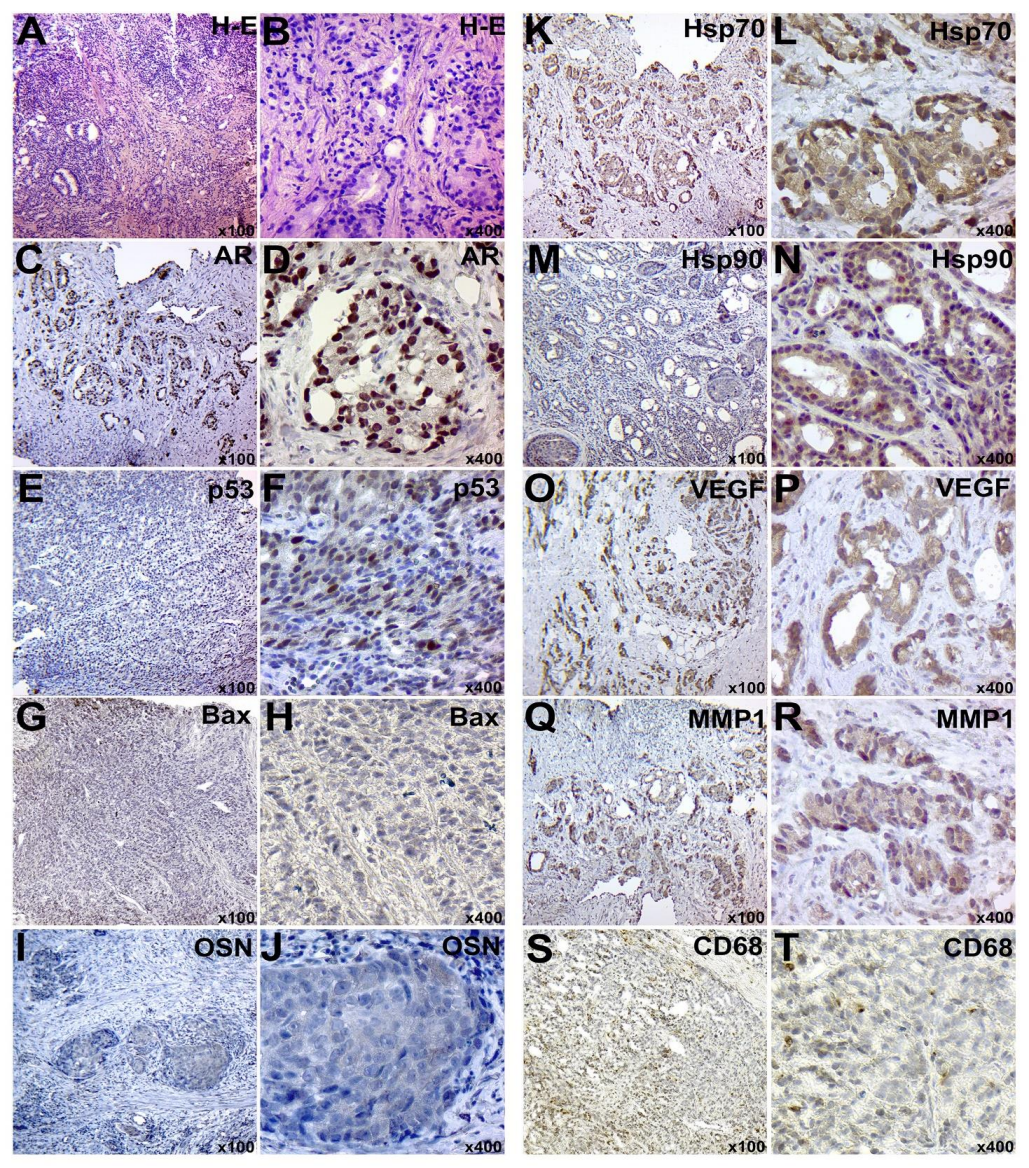
IHC examination of PC in subgroup C3: A-B – staining with hematoxylin and eosin; C-D – IHC detection of AR; E-F - IHC identification of p53 protein; G-H – IHC detection of p53; I-J – IHC of OSN expression; K-L – IHC detection of Hsp70; M-N – IHC detection of Hsp90; O-P – IHC detection of VEGF; Q-R – IHC detection of MMP-1; S-T – IHC detection of CD68+ positive cells. Chromogen – diaminobenzidine. Nuclei were counterstained with Mayer’s hematoxylin. The magnification is indicated in the lower right corner of each image

The tissue samples of both experimental and control groups had the inflammatory infiltration around tumor glands. However, the severity of the inflammatory process was higher in the experimental group. Inflammatory infiltration consisted mainly of macrophage cells, lymphocytes, and neutrophils (Figs. 1a, 1b, 2a, 2b, 3a, 3b, 4a and 4b).

IHC Characteristic of Tumor Tissue

IHC examination of AR expression in the PC tissue of subgroups E2 and E3 showed a clear nuclear reaction in tumor cells and in single cells of the peripheral tumor stroma (Figs. 1c, 1d, 2c, 2d and 6). The number of positive cells for the subgroup E2 corresponded to 396.79±26.02, and this number was 360.91±36.87 tumor cells for the subgroup E3 in the view field. The number of positive nuclei was 417.17±39.61 and 424.88±53.76 cells in the view field for samples of subgroups C2 and C3, respectively (Figs. 3c, 3d, 4c, 4d and 6).

The expression level of p53 protein in tumor tissue samples of subgroups E2 and E3 was 33.09 ± 3.3 and 46.7±2.15 cells in the view field, respectively (Fig. 1e, 1f, 2e, 2f and 6). The staining had a nuclear nature and positive cells were placed mainly in small groups. Moreover, 30.49±2.96 and 42.48 ± 3.36 tumor cells in the view field were detected in PC of subgroups C2 and C3, respectively (Figs. 3e, 3f, 4e, 4f and 6).

Pro-apoptotic Bax protein had a cytoplasmic nature of expression and moderate signal intensity. In PC samples of the subgroups E2 and E3, a total of 65.18 ± 3.53 and 58.05±3.79 positive cells were identified, respectively (Fig. 1g, 1h, 2g, 2h and 6). Meanwhile, the levels of expression corresponded to 42.69 ± 3.64 and 46.54±4.68 tumor cells in the view field in the subgroups C2 and C3, respectively (Fig. 3g, 3h, 4g, 4h and 6).

Hsp70 had a mixed nuclear-cytoplasmic nature of expression. The number of Hsp70-positive tumor cells in the tissues of subgroups E2 and E3 was 325.57±20.53 and 298.56 ± 17.19 cells, respectively (Fig. 1k, 1l, 2k, 2l and 6). In addition, the number of positive tumor cells in subgroups C2 and C3 was 274.73±32.72 and 260.72±17.7, respectively (Figs. 3k, 3l, 4k, 4l and 6).

During the IHC examination of the Hsp90, its expression was detected in the nucleus and cytoplasm of neoplastic cells. The number of positive elements was 352.84±26.39 in the subgroup E2 and 331.28 ± 28.5 in the subgroup E3 (Figs. 1m, 1n, 2m, 2n and 6). For subgroups C2 and C3, these levels corresponded to 413.3±39.66 and 454.98±17.42 cells (Figs. 3m, 3n, 4m, 4n and 6); also, its presence was detected between the corpora amylacea layers and the stagnant gland secretion.

VEGF had the cytoplasmic nature of the expression in the form of grains and lumps. In subgroups E2 and E3, the number of VEGF-positive tumor cells was 50.65±3.91 and 50.97±2.86, respectively (Figs. 1o, 1p, 2o, 2p and 6). The tumor tissue of subgroups C2 and C3 showed 68.84±4.8 and 54.08±2.09 positive neoplastic cells, respectively (Figs. 3o, 3p, 4o, 4p and 6). 

MMPI-1 was detected in the form of diffuse cytoplasmic expression. It was expressed in the both tumor and stromal components of PC. The number of positive tumor cells was 32.49 ± 1.6 and 43.92±1.82 in the tissues of subgroups E2 and E3, respectively (Fig. 1q, 1r, 2q, 2r and 6). In subgroups C2 and C3, 32.54±2.46 and 41.13±2.45 MMP1-positive cells were detected, respectively (Figs. 3q, 3r, 4q, 4r and 6).

CD68 had a cytoplasmic nature of expression. In tumor tissue and stroma of subgroups E2 and E3, a total of 138.11±5.57 and 136.86±4.05 CD68-positive cells were detected (Fig. 1s, 1t, 2s, 2t and 6); this number was 85.6±5.22 and 98.7 ± 6.09 positive cells for subgroups C2 and C3, respectively (Figs. 3s, 3t, 4s, 4t and 6).

The glycoprotein OSN had a diffuse cytoplasmic expression in tumor cells. The PC samples of subgroups E2 and E3 had 102.03±5.05 and 53.11±4.59 positive tumor cells, respectively (Figs. 1i, 1j, 2i, 2j and 6). Furthermore, subgroups C2 and C3 had a total of 43.1±3.44 and 30.5±2.88 OSN-positive neoplastic cells, respectively (Figs. 3i, 3j, 4i, 4j and 6).

**Figure 5:**
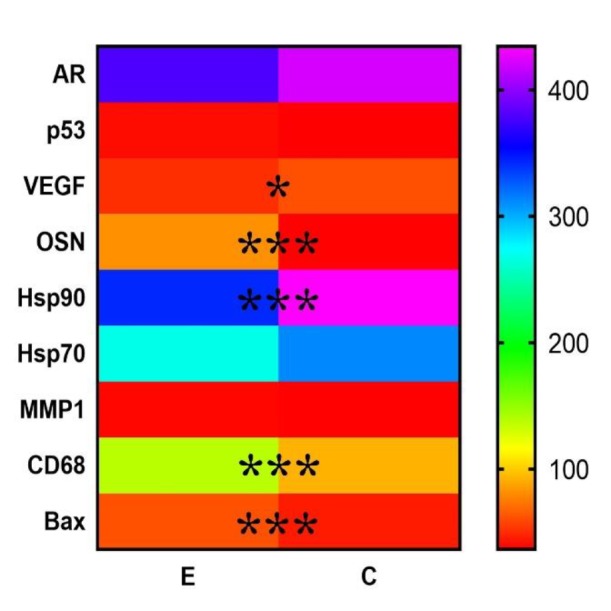
Mean values of AR, p53, VEGF, OSN, Hsp90, Hsp70, MMP-1, CD68, and Вах expression in the PC tissues. * – P<0.05, *** – P<0.001

The expression of the markers in groups E and C are presented in figure 5.

**Figure 6:**
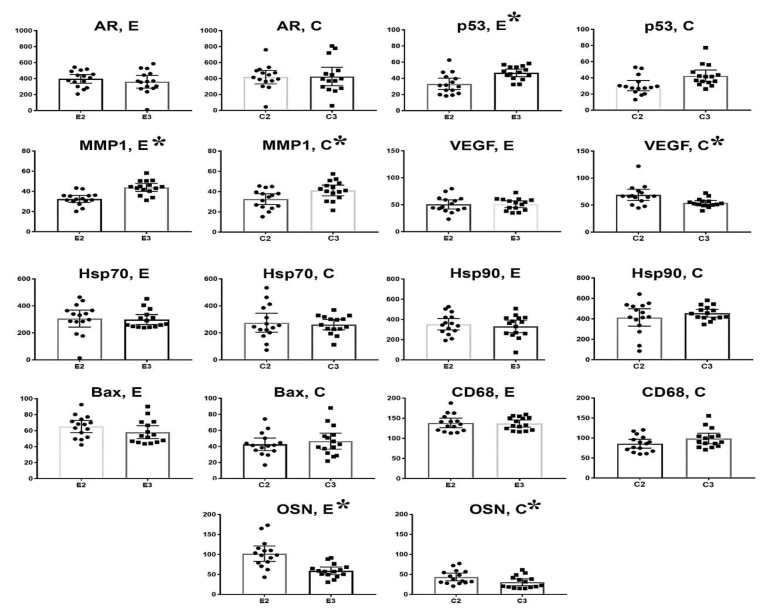
Statistical analysis of AR, p53, MMP-1, VEGF, Hsp70, Hsp90, Bax, CD68, and OSN expression in the experimental and control groups of PC tissues, depending on the grade score. The column indicates the mean value; the bar – 95% confidence interval, * – significant difference (P<0.05)

## Discussion

The average age of patients with PC in experimental and control groups was 70.23 ± 1.71 and 68.27 ± 1.27 years (*P*> 0.05), respectively. All PC samples were represented by acinar adenocarcinoma. 

During morphological examination of the PC tissue in the experimental group, prostate tissue structural transformation was manifested with cystic expansion of the glands, a retention of secretion, associated with the presence of IIn of amyloid and mineral nature. There were also the signs of chronic inflammation, mechanical injury of the glands epithelial component, the presence of cellular detritus in the lumen, glands micro-fractures and deformation (12). Also, fibrotic fibers growth, isolated hemorrhages, and moderate edema in the surrounding tissues were observed.

The neoplastic tissue of the control group samples was characterized by the IIn absence. This caused a decrease of the stagnant phenomena in the glands and ducts, and reduced the glandular epithelium traumatization. These changes led to a low intensity of inflammatory reaction in the PC tissue (13).

PC samples of E2 subgroup were characterized by a distinct structure of the epithelial component with the formation of well-recognized glands and pseudoglandular structures, by the production and accumulation of secretion. Secondary changes were not significant in tumor tissue.

Subgroup E3 was presented by PC tissue samples with a simplified structure of neoplastic tissue. Cancer cells were located in the form of unorganized mass, nests, chains, layers, and single cells. The simplicity of PC tumor tissue manifested with the monomorphic structure of tumor cells with significant decrease of glandular structures. The presence of secondary changes as necrosis, mucinous transformation, and hemorrhages were observed.

The characteristics of the PC samples of subgroup C2 included: maturity of atypical glandular structures, IIn absence, and secretion stagnation in the glandular-duct system. The structure of PC samples of subgroup C3 corresponded to the morphological structure of subgroup E3 (except the IIn presence and the inflammatory infiltration intensity).

During the IHC study of the androgens effect on the PC tissues, both groups showed a high sensitivity to these hormones in the absolute majority of the studied samples (1 sample with a negative reaction in the subgroup E3 was detected). There was no significant difference between AR expression of the groups (Fig. 5 and 6). In a previous study, the correlation between AR expression and functional activity of the neoplastic tissue and grade score was noted (14). However, our analysis of the influence of tumor grade score on the AR expression level indicated the absence of any significant difference in the rates of experimental and control groups. According to James L. Mohler (2008), changes in the genes encoding the androgen receptors, as well as the conformational changes in the structure of the AR itself, have a limited role in the PC progression and castration-resistant PC development. It was also found that the expression of androgen receptors does not depend on the grade score of tumor (15). 

System of pro-apoptotic proteins p53 and Bax has an important role in apoptotic processes. The p53-dependent apoptosis of the cells occurs indirectly by activating the Bax protein (Bcl-2 protein family) (16). The significant difference of p53 expression was found in subgroups E2 and E3 with a higher activity in high-grade PC (Fig. 6). These results may indicate a great dependence between the p53 expression and grade score, and a low correlation of this parameter with the presence of IIn. These results are in full agreement with the findings of the study conducted by Thomas DJ et al. (1993), in which they found that high expression of p53 is inherent predominantly for high-grade tumors and is accompanied by a deterioration in the prognosis of the disease and reduced patients life expectancy (17). 

Bax expression analysis demonstrated a significant difference between the experimental and control groups. But there was no statistically significant difference in the results of the IHC reaction between subgroups E2 and E3, as well as C2 and C3. This suggests that IIn stimulate the pro-apoptotic proteins formation and promote apoptosis, while the role of the grade score in the Bax expression was not proven (Fig. 5). However, Li-Yan Khor (2006) indicated the direct dependency of the Bax expression to the Gleason score and prognosis and concluded that more aggressive or high-grade tumors have an increased Bax expression (18-19). 

The role of the Hsp70 and Hsp90 in the apoptosis inhibition has been proven. These proteins inhibit the activity of pro-apoptotic proteins (in particular, caspases) (20). In our study, a statistically significant difference was found between the expression of Hsp90 in the experimental and control groups (Fig. 5). The presence of Hsp90 in the structure of corpora amylacea was detected due to their probable participation in the formation of these inclusions. No correlation was found between the grade score and Hsp90 expression (Fig. 6). During the study of the Hsp70 expression, there was no significant difference between the experimental and control groups, subgroups E2 and E3, and C2 and C3. However, there was a tendency toward their increase in PC with the presence of IIn. This suggests that neither the PC grade score nor the presence of IIn can influence the expression of Hsp70 (Fig. 5 and 6). Although majority of studies point to the insignificant role of high-molecular heat shock proteins in the processes of tumorigenesis (21), their association with the risk of metastatic breast cancer development was found (22). There is also a link between the p53 and Hsp70 (inhibits the caspase-9-dependent mitochondrial apoptosis, stabilizes the structure of the mutant p53 protein, and stimulates cell proliferation). Hsp70 is associated with a more aggressive course of the disease and is an adverse prognostic factor (23). Meanwhile, the dependence of the Hsp90 expression on the presence of IIn is unexplored. However, it has been found that elevated levels of extracellular Hsp90 increase cellular migration, invasive potential of PC models, and tumorigenicity (24). 

Expression of MMP-1 is associated with a tumor invasion and development of metastases. That is why the expression of MMP-1 in tumor tissue is considered as unfavorable factor for PC prognosis. (25). There was no statistically significant difference between the experimental and control groups during the IHC analysis (Fig. 5). However, we detected the effect of PC grade score on the MMP-1 expression in both experimental and control groups. Moreover, a significantly higher MMP-1 expression was found in the high-grade PC (E3 and C3) (Fig. 6). These findings are supported by the results of Zhong et al. (2008), in which they indicated the correlation between the expression of MMP-1 and the disease stage (26). MMP-1, or collagenase 1, is involved in the destruction of the connective tissue component of the tumor stroma and creates conditions for the development of PC metastases (27). Higher levels of MMP-1 expression in high-grade tumors of both groups can be explained by this phenomenon.

Increased expression of VEGF by tumor tissue is considered to be a prognostic-adverse factor (28). Our results indicated a significantly higher expression of VEGF in the control group (Fig. 5). The prevalence of expression in subgroup C2 was found in comparison to subgroup C3. This may indicate a negative influence of IIn on tumor tissue vascularization processes. However, most studies indicate an increased VEGF expression in high-grade tumors (29-30). 

We used IHC for the evaluation of CD68 expression to determine the number of macrophage cells. A significantly higher number of CD68-positive cells was found in the experimental group (Fig. 5). There was no difference in the number of CD68-positive cells in tumors with a different grade score. This indicates that the grade score does not influence the infiltration of tumor tissue by macrophages. M. Lanciotti et al. (2014) found that the increased number of tumor activated macrophages is associated with the extracapsular extension and progression of PC (31). Thus, the significant number of the mononuclear phagocyte cells in PC tissues of experimental group indicates the role of IIn in the chronic inflammation development. 

OSN is involved in the processes of biomine-ralization and remodeling of the extracellular matrix; and it may be a tumor growth inhibitor. Its level of expression depends on the grade score of the epithelial and stromal components of various tumor types (32). During the study, we found a significantly higher level of OSN expression in the PC samples of the experimental group (Fig. 5). This may be stipulated by his participation in the prostatic calculi formation and growth. There was also a predominance of OSN expression in the moderate-grade PC tissues of both groups (*P*<0.05) (Fig. 6). According to K. Jacob et al. (1999), OSN specifically enhances the activity of matrix metalloproteinases and promotes the migration of PC cells and the development of bone metastases (33). Therefore, we suggest that increased OSN expression is associated with the presence of IIn and increased expression of MMP-1. The combination of these factors contributes to the development of metastases.

## Conclusion

The presence of IIn in the PC tissues of experimental group promotes tissue remodeling with a violation of the glands’ secretory cycle and drainage function. These changes lead to mechanical traumatism, chronic inflammation development, and fibrosis.

In the PC samples, a high AR expression was detected, which did not depend on the grade score and the presence of IIn.

The level of VEGF expression was higher in low-grade PC with IIn. This may indicate a suppressive effect of IIn on the vascularization of tumor tissue. The significance of this phenomenon is unclear and requires further research.

With a decrease in the grade score of PC, expression level of prognostic-adverse markers p53 (in the case of the IIn presence) and MMP-1 increases.

The OSN expression is higher in PC tissues with IIn due to its participation in the processes of biomine-ralization. Meanwhile, the expression of this glycoprotein is lower in the high-grade PC.

The number of CD68-positive and Bax-positive cells is significantly higher in the PC with IIn. This may be determined by physical injuries of the epithelium and surrounding tissues with the development of chronic inflammation.

The Hsp90 has a significantly lower expression in the PC tissues with IIn, which may be determined by its deposition in the structure of corpora amylacea.

Expression of the Hsp70 was independent of the presence of IIn and on the PC grade score.
